# CD80 and CD86 Differentially Regulate Mechanical Interactions of T-Cells with Antigen-Presenting Dendritic Cells and B-Cells

**DOI:** 10.1371/journal.pone.0045185

**Published:** 2012-09-14

**Authors:** Tong Seng Lim, James Kang Hao Goh, Alessandra Mortellaro, Chwee Teck Lim, Günter J. Hämmerling, Paola Ricciardi-Castagnoli

**Affiliations:** 1 Singapore Immunology Network, Agency for Science, Technology and Research (A*STAR), Singapore, Singapore; 2 Mechanobiology Institute, National University of Singapore, Singapore, Singapore; 3 Department of Bioengineering & Department of Mechanical Engineering, National University of Singapore, Singapore, Singapore; 4 Division of Molecular Immunology, German Cancer Research Center DKFZ, Heidelberg, Germany; Institute of Microbial Technology, India

## Abstract

Functional T-cell responses are initiated by physical interactions between T-cells and antigen-presenting cells (APCs), including dendritic cells (DCs) and B-cells. T-cells are activated more effectively by DCs than by B-cells, but little is known about the key molecular mechanisms that underpin the particular potency of DC in triggering T-cell responses. To better understand the influence of physical intercellular interactions on APC efficacy in activating T-cells, we used single cell force spectroscopy to characterize and compare the mechanical forces of interactions between DC:T-cells and B:T-cells. Following antigen stimulation, intercellular interactions of DC:T-cell conjugates were stronger than B:T-cell interactions. DCs induced higher levels of T-cell calcium mobilization and production of IL-2 and IFNγ than were elicited by B-cells, thus suggesting that tight intercellular contacts are important in providing mechanically stable environment to initiate T-cell activation. Blocking antibodies targeting surface co-stimulatory molecules CD80 or CD86 weakened intercellular interactions and dampen T-cell activation, highlighting the amplificatory roles of CD80/86 in regulating APC:T-cell interactions and T-cell functional activation. The variable strength of mechanical forces between DC:T-cells and B:T-cell interactions were not solely dependent on differential APC expression of CD80/86, since DCs were superior to B-cells in promoting strong interactions with T-cells even when CD80 and CD86 were inhibited. These data provide mechanical insights into the effects of co-stimulatory molecules in regulating APC:T-cell interactions.

## Introduction

Adaptive immune responses are initiated by specific interactions between T-cells and antigen-presenting cells (APCs). T-cell activation involves the formation of specialized structures at areas of APC:T-cell intercellular contact, which have been termed immunological synapses (IS) [1], [2], [3], [4], [5]. The shape and structure of IS on the T-cell surface are influenced by encountering with different types of APC, including dendritic cells (DCs) and B-cells [6]. While resting B-cells induce the formation of a singular, mature IS, multifocal IS have been observed between T-cells and DCs [4], [7], [8]. In addition to inducing distinct IS conformations, DCs and B-cells also differ in their expression of cell-surface adhesion molecules [9], [10], [11], [12], as well as their surface morphology and cytoskeletal dynamics [10], [13]. While these distinct characteristics have been convincingly shown to impact on the ability of APC subsets to regulate T-cell activation [4], [7], [8], [9], [10], [11], [12], [13], [14], [15], [16], [17], studies on the role of biophysical interactions between T-cells and APCs remain limited.

Previous reports have demonstrated that the duration of cell:cell interactions is inversely correlated with APC potency in activating T-cells [10], [13], [15], [16], [18]. Indeed DC:T-cell interactions are both more dynamic and more potent in inducing T-cell activation when compared with the long contact duration that occurs between T-cells and resting B-cells. These studies have provided novel insights into the temporal dynamics of biophysical interactions in IS, but so far there has been no systematic analysis of the mechanical strength of interactions between T-cells and different type of APCs, and the consequences of these interactions for T-cell activation.

We have previously shown that immune synapse formation determines the interaction forces between T-cells and B-cells [19]. Moreover, we have shown that the mechanical interactions between T-cells and DCs correlate with T-cell functional responsiveness [20]. In the current report, we have used antigen-specific T-cells that specifically recognize ovalbumin-derived peptide [21], [22] combined with single cell force spectroscopy (SCFS) [19], [20], [23], [24] to compare and characterize the mechanical force of T-cell interactions with DCs and B-cells. Our data indicate that upon stimulation with antigenic peptides, DC:T-cell interactions were far stronger than B:T-cell interactions. Stronger DC:T-cell interactions were associated with more efficient T-cell activation, as assessed by elevated calcium mobilization and higher secretion levels of cytokines IL-2 and IFN-γ. Dampened T-cell activation was associated with the weakened APC:T-cell interactions when blocked by using antibodies targeting co-stimulus molecules CD80 and CD86, suggesting that CD80 and CD86 are important in strengthening intercellular interactions and amplifying T-cell functional activation. However, DC:T-cell interactions still remained stronger than B:T-cell interactions despite inhibition of co-stimulatory molecules CD80 and CD86, indicating that the variable strength of mechanical forces between DC:T-cells and B:T-cell interactions were not solely contributed by the differential APC expression of co-stimulatory molecules CD80 and CD86. Taken together, these data provided mechanical insights into the roles of co-stimulatory molecules in regulating intercellular APC:T-cell interactions.

## Materials and Methods

### Mice

OT-I.Rag1-/- mice [22], [25] were provided by Taconic from the National Institute of Allergy and Infectious Diseases Exchange Program (# 004175; Bethesda, MD) and maintained at the SPF animal facility of the Biological Resource Centre (BRC) of Biopolis in Singapore.

### Ethics Statement

This study was carried out in strict accordance with the recommendations in the Guide for the Institutional Animal Care and Use Committee (IACUC) of the Biological Resource Centre (BRC) of Biopolis in Singapore. The BRC IACUC protocol was approved by the National Advisory Committee for Laboratory Animal Research in Singapore (Permit Number: 110626).

### Cells, Peptides and antibodies

The splenic D1 dendritic cell line [26], and the B-cell hybridoma LB27.4 [27], which expresses MHC class-I H-2k^b^ and is able to present Ova-peptide SIINFEKL were used in these analyses. Splenic CD8+ T-cells were purified from OT-I mice by negative selection using CD8α+ T-cell isolation kit II (Miltenyi Biotec) according to the manufacturer's protocol. Freshly isolated OT-I T-cells were maintained at 37°C in endotoxin free Iscove's Modified Dulbecco's Medium (IMDM, EUROCLONE) containing 10% fetal bovine serum (100 IU/ml Penicillin, 100 µg/ml Streptomycin, 2 mM Glutamine, and 50 µM β-mercaptoethanol (all from GIBCO) before use in atomic force microscopy experiments. Ova Peptides (SIINFEKL) were purchased from Anaspec Corporation.

### Flow Cytometry

DCs or freshly isolated OT-I T-cells were stained with antibodies against cell surface molecules and were measured by BD FACSCalibur flow-cytometer. Antibodies: biotinylated anti-mouse CD43 (S7), FITC-conjugated murine (BALBC/c) anti-mouse H-2K^b^ (AF6-88.5), hamster anti-mouse CD11c-APC (HL3), rat anti-mouse CD19-PE (1D3), hamster anti-mouse CD54-FITC (3E2), hamster anti-mouse CD80-PE (16-10A1), rat anti-mouse CD86-PE (GL1) and anti-mouse Ova-H-2K^b^ (25-DC.16, eBioscience). Isotype controls: FITC mouse IgG2a κ (G155-178), APC hamster IgG1, λ1 (G235-2356), PE rat IgG2a, κ (R35-95), FITC hamster IgG1 κ (A19-3) and PE hamster IgG2, κ (B81-3) (all from BD PharMingen).

### Functionalization of Atomic Force Microscopy (AFM) Cantilevers

Functionalization of AFM cantilever was performed as previously described [19], [20]. Briefly, soft tip-less silicon nitride tips (NP-O10, Veeco, Santa Barbara, CA) with a nominal spring constant of 0.06 N/m were coated with biotinylated BSA (0.5 mg/ml in 0.1 M NaHCO_3_, Sigma) over night at 37°C. After washing three times with PBS, the tips were incubated in 0.5 mg/ml streptavidin (Sigma-Aldrich) for 1 h. Cantilevers were washed again and incubated with 0.5 mg/ml of biotinylated anti-CD43 antibody (BD PharMingen) for 1 h. Prior to each experiment, the spring constant of the cantilever was determined using the built-in thermal tune module of the AFM.

### Single Cell Force Spectroscopy

The basic principles of single cell force spectroscopy using AFM have been described elsewhere [19], [20], [23], [24]. AFM measurements were performed with a MultiMode™ Picoforce™ AFM (Veeco) coupled to a microscope using fluid cell-in-cell culture medium at 37°C on a heated plate. One day before the experiment, APCs were seeded onto round cover slips and incubated overnight to allow firm adhesion. Non-adherent cells were washed away and not used for Single Cell force Spectroscopy. APCs were pulsed or not with peptides 4 h before the AFM experiment. Peptide-pulsed APCs were maintained in the continuous presence of the same concentration of Ova peptides in cell culture medium without washing. Freshly isolated T-cells were attached to the anti-CD43 functionalized cantilever. Force-distance curves were obtained by positioning the cantilever with the attached OT-I T-cell onto the adherent APC on the cover slip and applying contact force of 1 nN with predefined contact duration. The retraction speed was set to 1 µm/s for all measurements in all conditions for comparative purposes. For long contact durations (3 minutes) a new T-cell was attached to the cantilever for each AFM measurement. At least 10 pairs of APC:T-cell interactions were probed for each experimental point. All data were analyzed with MATLAB (The MathWorks, Natick, MA) to quantify the interaction force of individual APC:T-cell conjugates.

### ELISA cytokine assay

DCs or B-cells were pre-pulsed for 4 h with different Ova peptide concentrations and were then co-cultured with OT-I T-cells in 96-well plates (5×10^4^ DC and 5×10^4^ T-cells per well) without washing. IL-2 and IFN-γ secretion were measured by ELISA after 24 h of co-culture.

### Calcium mobilization

Freshly isolated OT-I T-cells were incubated with 1 µM Fluo-4-AM and 10 µM Fura-Red-AM (Invitrogen) at 37°C for 1 h in cell culture medium. After washing twice with medium, the T-cells were allowed to bind to DCs or B-cells grown on glass-bottom dishes (MatTek). Calcium responses in individual T-cells were measured using an inverted microscope (Olympus IX81) with a 60X objective. The T-cells were illuminated at 488 nm and the fluorescent emission of Fluo-4-AM and Fura-Red-AM was captured every 5 s by time-lapse confocal imaging (Olympus FV1000) and then analysed with Imaris software (Bitplane). Integrated Fluo-4/Fura-Red ratio was calculated from fluorescent images as a measurement for intracellular calcium concentration.

### Blocking of CD80 and CD86

For blocking experiments, APCs were incubated with 10 µg/ml antibodies against CD80 (16-10A1) and/or CD86 (GL1) for 30 min at 37°C prior to AFM experiments, or for 24 h prior to the ELISA cytokine assays. Hamster IgG2 κ (B81-3) and rat IgG2a κ (R35-95) were used as isotype controls in the blocking experiments. All AFM and ELISA cytokine measurements were performed in the continuous presence of the same concentration of the blocking antibodies in cell culture medium.

## Results

### Dendritic Cells and B-cells Express Comparable Levels of MHC Class I

**Figure 1:**
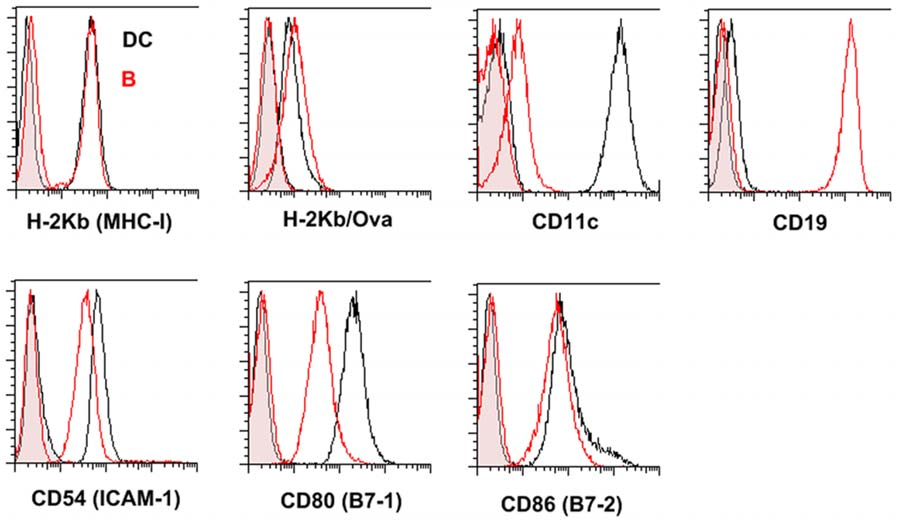
B-cells and DCs exhibit a similar phenotype. B-cells (Red) and DCs (Black) were stained with antibodies against H-2K^b^ (MHC class I), H-2K^b^/Ova (pMHC), CD11c, CD19, CD54 (ICAM-1), CD80 (B7-1) and CD86 (B7-2). Filled histograms: isotype controls; Unfilled histograms: staining with antibody. Data are representative of three independent experiments.

To directly compare DC:T-cell and B:T-cell interactions, we used the well characterized D1 cells derived from splenic DCs [26], alongside B-cell hybridoma LB27.4 [27] which expresses MHC class-I H-2K^b^ and can present antigenic ovalbumin peptide Ova _257–264_ (SIINFEKL) to OT-I T-cells [21]. FACS analysis confirmed that the purity of DCs (CD11c+, H-2kb+) and B-cells (CD19+, H-2K^b^ +) was >98%, allowing us to investigate DC:T-cell and B:T-cell interactions at the single cell level. Expression of surface markers CD54 (ICAM-1), CD80 (B7-1) and CD86 (B7-2) are shown in Fig. 1. B-cells expressed the same surface level of MHC-I and CD86 as DC, but displayed slightly lower levels of ICAM-1 and CD80. In addition, Ova peptide could be loaded successfully onto both DCs and B-cells, as determined by staining with an antibody against Ova-bound H-2K^b^ (H-2K^b^/OVA, Fig. 1).

### T-cell Activation is Promoted More Efficiently by DCs than by B-cells

**Figure 2:**
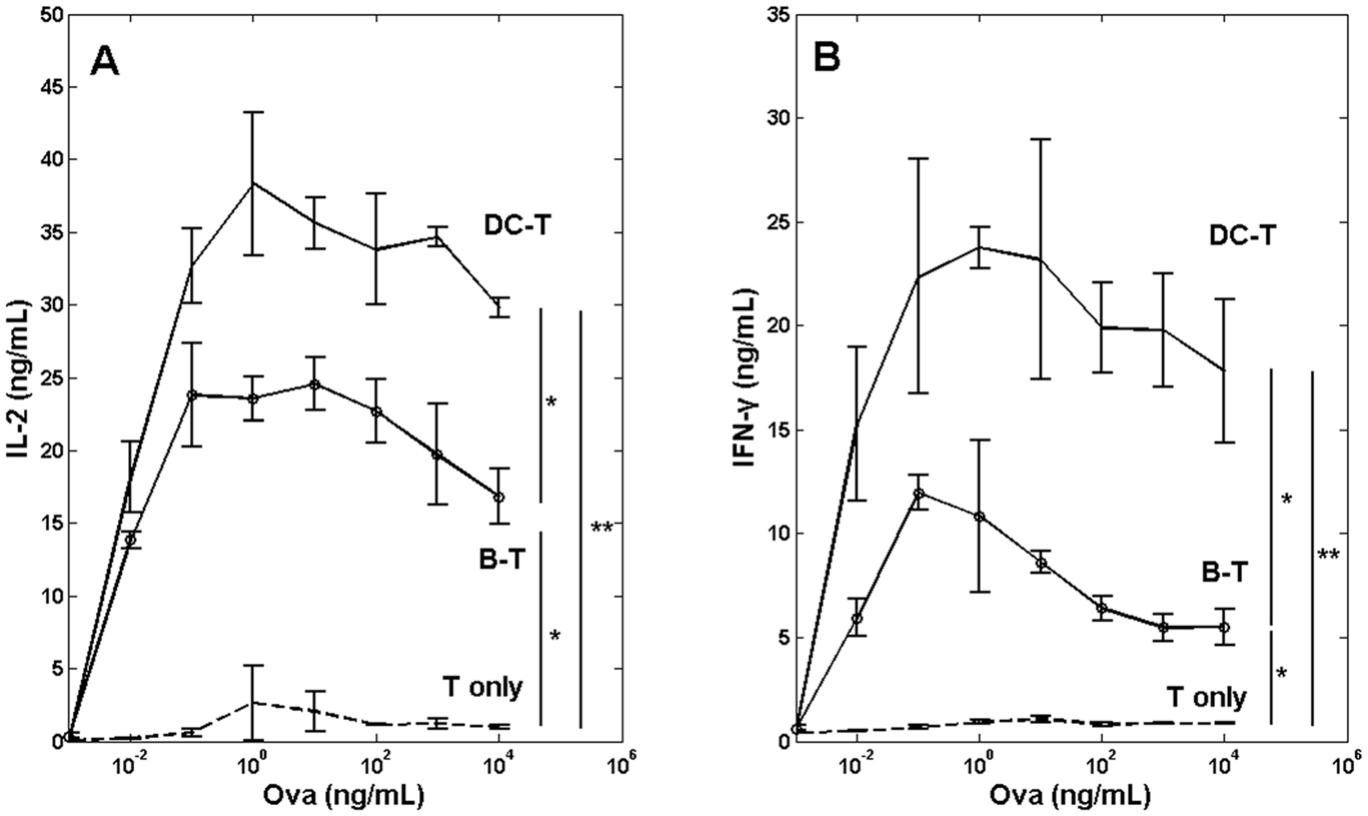
Cytokine secretion by DC:T-cell and B:T-cell co-cultures. (**A**) and (**B**): secretion of IL-2 (left) and IFN-γ (right) after 1 day co-culture of CD8+ OT-I T-cells with DCs or B-cells pre-pulsed or not with Ova peptide (10 pg–10 µg/ml). *p<0.01; **p<0.001, unpaired *t*-test. Bars indicate mean ± s.e.m. Data are representative of three independent experiments.

To evaluate how T-cell activation was influenced by different antigen-loaded APC subsets, we measured cytokine secretion upon T-cell stimulation by DCs or B-cells pre-pulsed with antigenic Ova peptides at different concentrations (10 pg-10 µg/ml). Fig. 2 shows that antigen-pulsed DCs activate OT-I cells more potently than B-cells presenting comparable amounts of peptide, as measured by release of IL-2 and IFN-γ.

**Figure 3:**
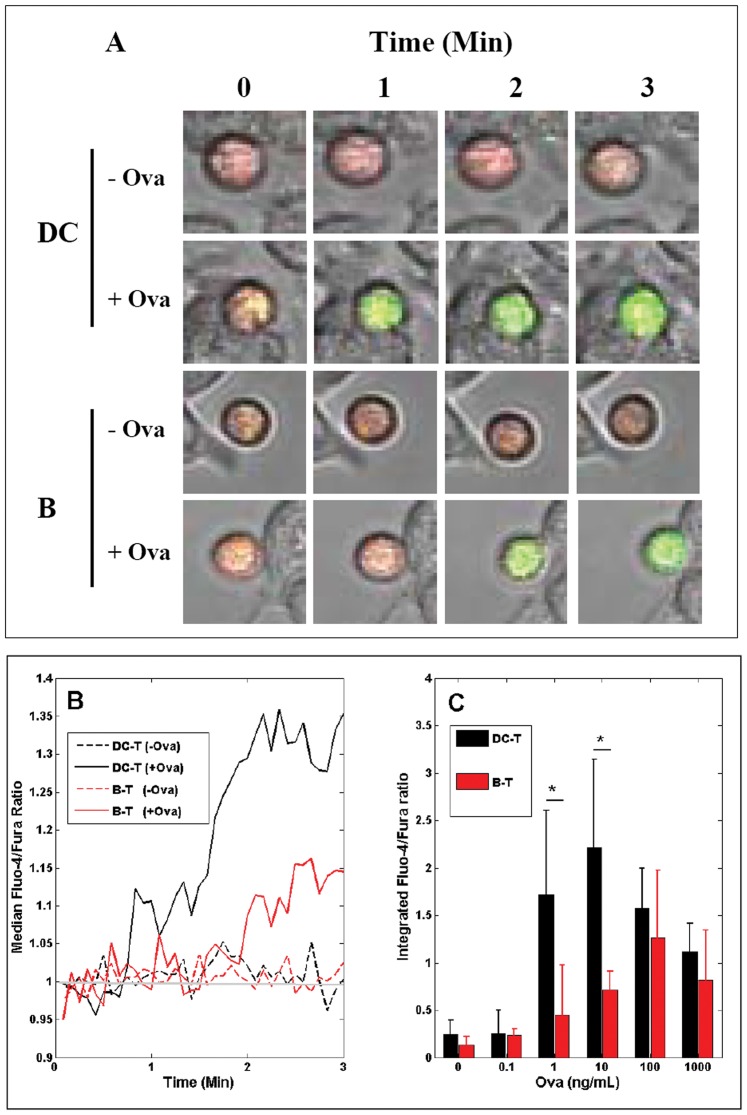
Calcium response of T-cells bound to APCs. (**A**) Representative differential interference contrast (DIC) images of the DC:T-cell or B:T-cell conjugates overlaid with the Fluo-4 (green) and Fura-red (red) fluorescent signals (loaded in T-cells only) at the indicated time points. DCs or B-cells were pre-pulsed or not with Ova peptides (10 ng/ml) for 4 h prior to co-culture with T-cells. (**B**) Time course of intracellular calcium concentrations in the responding T-cells, as measured by Fluo-4/Fura-red ratio. Each plot represents data from a pair of cell:cell conjugates. (**C**) Average calcium response of T-cells bound to DC (black) or B-cell (red) pre-pulsed with Ova peptides at different concentrations (0.1 ng–1 µg/ml). To quantify early calcium response in T-cells, Fluo-4/Fura-red ratios were measured every 5s in responding T-cells and were then integrated for 3 min from the time of the initial calcium increase (grey line, Fig 4B). For each condition, the average calcium response was measured by pooling data of APC:T-cell conjugates (n>15 pairs of cells) from >3 independent experiments. Bars indicate mean ± s.e.m. *p<0.01, unpaired *t*-test.

To measure T-cell activation at earlier time points we also assayed the calcium response of OT-I T-cells after stimulation with peptide-pulsed DCs or B-cells. Fig. 3A shows representative calcium responses of T-cells bound to DC or B-cells pre-incubated with Ova peptide (10 ng/ml). In the absence of peptide, the calcium signal in DC:T-cell and B:T-cell conjugates remained low, as indicated by a low Fluo-4/Fura ratio. In contrast, intracellular calcium was rapidly mobilized in the presence of Ova peptide, and calcium levels progressively increased during the 3 min observation period (Fig. 3B). To directly compare the calcium responses of T-cells bound to the different types of APC, we integrated the Fluo-4/Fura ratio [20], [28] of cell:cell conjugates for a contact duration of 3 min (Fig. 3B). The average calcium responses of T-cells after exposure to APCs pre-pulsed with Ova peptides are shown in Fig. 3C.

**Figure 4:**
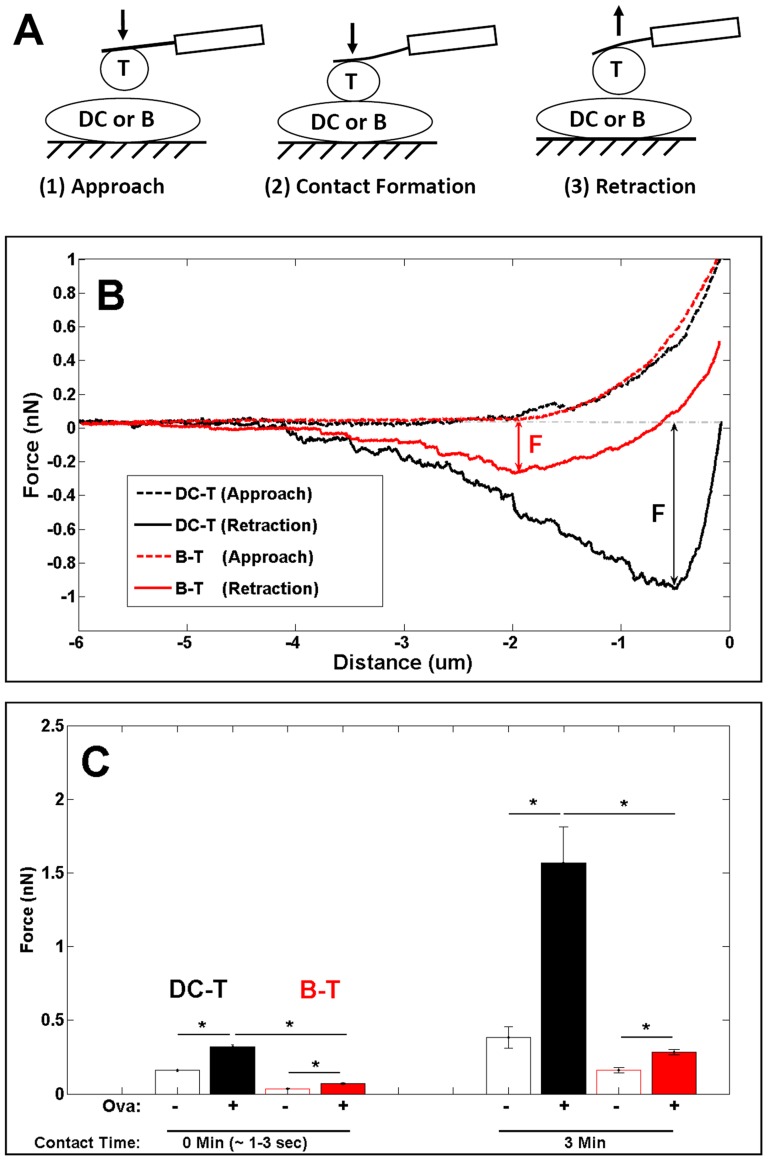
Differential Strength of Mechanical Forces between APC:T-cell Conjugates. (**A**) Schematic illustration of AFM experiments. A T-cell-mounted AFM cantilever was placed above a DC or B-cell that was firmly attached to a glass cover slip. The T-cell was then brought into contact with the target cell. Interaction forces were measured by the deflection of the cantilever after a pre-defined contact time. (**B**) Corresponding force-distance curve of DC:T-cell (black) or B:T-cell (red) interactions. F represents maximal interaction force (double arrow). (**C**) Interaction forces of DC:T-cell (black) or B:T-cell conjugates (red) in the absence (-Ova) or presence of Ova peptide (+Ova) for contact time of ∼1–3 sec and 3 min. *p<0.01, unpaired *t*-test. Bars indicate mean ± s.e.m (n>10 pairs of cells). For each condition, OT-I T-cells were isolated from >3 independent experiments.

The magnitude of the calcium responses in the APC:T-cell conjugates varied according to the concentration of peptides pulsed onto the APCs, but at a constant peptide concentration, DCs were superior to B-cells at inducing calcium responses in T-cells. In addition, the threshold peptide concentration required by DCs to trigger optimal calcium responses in T-cells (10 ng/ml) was 10-fold less than that required by B-cells (100 ng/ml; Fig. 3C). Increasing peptide pulse concentrations beyond these threshold levels resulted in impaired calcium responses in the stimulated T-cells (Fig. 3C).

### Differential Strength of Mechanical Forces between APC:T-cell Conjugates

We used single cell force spectroscopy (SCFS) to investigate the mechanical force of DC:T-cell and B:T-cell interactions (Fig. 4A). To quantify the strength of these mechanical interactions, the maximal interaction force (F) [19], [20], [23], [29] was determined for individual cell:cell conjugates (Fig. 4B). The average interaction force was used to compare interactions between DC:T-cell and B:T-cell in response to antigen stimulation.

The interaction forces of DC:T-cell (black bar) in the absence and presence of Ova peptide (10 ng/ml) are shown in Fig. 4C. We observed that the DC:T-cell interaction force in the presence of Ova peptide after ∼1–3s contact duration (average interaction force 0.32±0.02 nN) was increased after 3 min contact duration (1.57±0.25 nN). In the absence of Ova peptide the interaction force was weak (<0.4 nN), highlighting the requirement for Ova peptide in establishing strong interaction forces between Ova-specific T-cells and DCs. B:T-cell interactions were also strengthened after antigen stimulation, exhibiting an interaction force of 0.07±0.01 nN after 1–3s, and 0.28±0.02 nN after a contact duration of 3 min (red bar, Fig. 4C). However, the B:T-cell binding forces (<0.4 nN) were significantly lower than was observed for DC:T-cell interactions (Fig. 4C), confirming that DCs are superior to B-cells in establishing tight interactions with antigen-specific T-cells.

### CD80 and CD86 Contribute to Strong DC:T-cell Interaction Forces

**Figure 5:**
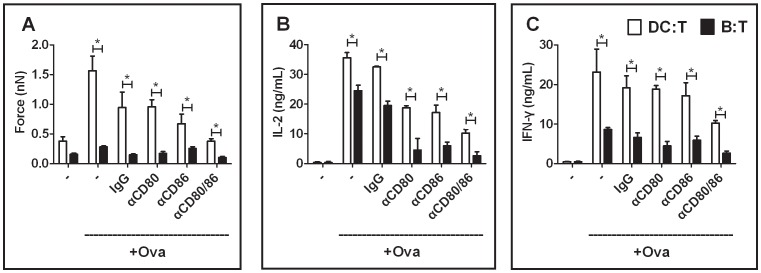
Contribution of CD80 and CD86 to Interaction Forces and Cytokine Secretion. (**A**) Interaction forces; (**B**) IL-2 and (**C**) IFN-γ production of DC:T-cell or B:T-cell conjugates in the presence of blocking antibodies against CD80 and/or CD86. DCs or B-cells were pre-pulsed with Ova peptides (+Ova, 10 ng/ml) before antibody blocking. All force measurements were conducted with contact time of 3 min. *p<0.01; unpaired *t*-test. For each condition, OT-I T-cells were isolated from >3 independent experiments. αCD80: blocking antibody targeting CD80; αCD86: blocking antibody targeting CD86; αCD80/86: αCD80 and αCD86 simultaneously; IgG: isotype controls for both αCD80 and αCD86.

We have previously shown that both TCR-pMHC and LFA-1/ICAM-1 are important adhesion molecules in promoting strong DC:T-cell interactions [20]. We therefore performed blocking experiments using antibodies targeting CD80 (B7-1) and CD86 (B7-2) molecules, which were expressed both on the surface of DCs and B-cells (Fig. 1). As shown in Fig. 5A, antibodies to CD80 and CD86 reduced the interaction forces of DC:T-cell conjugates to 0.96±0.12 nN and 0.67±0.16 nN respectively. When CD80 and CD86 were blocked simultaneously, the average interaction force of DC:T-cell conjugates was decreased further (0.38±0.04 nN). However, B:T-cell interactions remained weaker than DC:T-cell interactions under all conditions, with interaction forces <0.4 nN in all cases (Fig. 5A).

To investigate whether the force reduction upon inhibition of CD80 and CD86 resulted in impaired T-cell activation, we measured IL-2 and IFN-γ release after antibody blockade of co-stimulation. Consistent with the force measurement data, both IL-2 and IFN-γ secretion were reduced in CD80 or CD86 blocking conditions. The reduction in cytokine release was most significant when both CD80 and CD86 co-stimulation were inhibited simultaneously (Fig. 5B, 5C), confirming the key role played by co-stimulatory molecules in functional T-cell activation.

## Discussion

We used single cell force spectroscopy to characterize and compare the mechanical force of DC:T-cell and B:T-cell interactions following antigen recognition. Our data reveal that DCs are superior to B-cells in establishing strong interactions with T-cells upon antigen stimulation. The differential strength of APC interactions with T-cells are complex, and cannot be explained solely by differences in MHC class I expression, or by differences in APC antigen loading, since both DCs and B-cells displayed similar levels of H-2K^b^/Ova complexes at the cell surface.

Cellular binding forces depend on the recognition of co-stimulatory molecules including CD80 and CD86. Accordingly, treatment with CD80/86 blocking antibodies reduced the interaction force of cell:cell conjugates. Both CD80 and CD86 can bind to the T-cell stimulatory receptor CD28 [30], [31], [32], [33], [34], and to the inhibitory receptor CTLA4 [35], [36], [37]. CD86 appeared to strengthen DC:T-cell interactions more markedly than CD80, since higher force reduction was observed after blocking CD86 alone than was achieved by disrupting CD80 alone. The ability of CD86 to induce stronger DC:T-cell interactions is consistent with the crucial role played by this molecule in initiating immune responses [38], [39]. However, DC:T-cell interaction forces could not be completely abrogated by single antibody blockade of CD80 or CD86, which could perhaps be due to the functional redundancy of these co-stimulatory markers [40], [41]. Differential surface expression of CD80/86 between DCs and B-cells cannot explain the differences in binding strength that we observed between DC:T-cell and B:T-cell interactions, since DCs were consistently superior to B-cells in establishing strong interactions with T-cells, even when subject to complete blockade of CD80 and CD86 molecules. However, there may be intrinsic functional differences in the signaling triggered by these co-stimulatory molecules between different APC populations, which could perhaps account for the functional differences observed between DC:T-cell and B:T-cell interactions [14].

T-cell activation is initiated by the immunological synapse (IS) formed at the intercellular contacts between APCs and antigen-specific T-cells. The IS has been demonstrated to be a dynamic complex that consists not only the TCRs, but also sets of co-stimulatory receptors and ligands [42], [43], [44], [45], [46], [47], [48], [49], [50], [51]. CD28, the most powerful T-cell co-stimulatory receptors for CD80 or CD86, has been shown to localize coordinately with TCRs to form microclusters that are able to recruit protein kinase C θ (PKCθ) to initiate T-cell activation [45], [52], [53]. In addition to the functional roles of modulating T-cell responses, CD28 may also modify morphological features of the IS [54], [55], [56]. Previously, we have shown that IS formation determines the interaction forces between T cells and APCs [19]. Here, APC:T-cell interactions were weakened by CD80 and/or CD86 blocking, which could directly inhibit CD28 signaling that stabilizes IS by promoting enlarged contact area between APC:T-cell conjugation [54], [57]. Alternatively, inhibition of CD28 signaling may interfere with the regulation of cytoskeletal signaling through the small guanosine triphosphate hydrolase (GTPase), Rac1 and/or cell division cycle 42 (Cdc42) [55], [56], or the accumulation of lipid raft or raft-localizing molecules [44], [46], [58], [59], [60] at IS. This could explain why force reduction was observed followed by the inhibition of CD28 signalling since cytoskeleton dynamics and membrane lipid rafts are important in promoting strong interactions between T-cells and APCs [61].

Our data provide novel insights into the amplificatory roles of co-stimulus molecules CD80 and CD86 in strengthening APC:T-cell interactions. As compared to B:T-cell interactions, stronger DC:T-cell interactions are likely to provide a mechanically stable environment that induces potent functional T-cell activation.
